# Resilience Coping in Preschool Children: The Role of Emotional Ability, Age, and Gender

**DOI:** 10.3390/ijerph18095027

**Published:** 2021-05-10

**Authors:** Huaruo Chen, Qiuyun Hong, Jie Xu, Fei Liu, Ya Wen, Xueying Gu

**Affiliations:** 1 School of Education Science, Nanjing Normal University, Nanjing 210046, China; 190601021@njnu.edu.cn (H.C.); ReginaHong@163.com (Q.H.); 190601022@njnu.edu.cn (J.X.); 190601009@njnu.edu.cn (F.L.); 170601026@njnu.edu.cn (Y.W.); 2 Center for Research and Reform in Education, Johns Hopkins University, Baltimore, MD 21286, USA; 3 School of Education Science, Huaiyin Normal University, Nanjing 210046, China; 4 School of Teacher Education, Nanjing Xiaozhuang University, Nanjing 210046, China

**Keywords:** emotional ability, emotional understanding, emotional regulation, resilience, Chinese preschool children

## Abstract

Background: In the process of children’s physical and mental development, emotional ability is an important part of their cognitive and social ability. Resilience in the face of difficulties or setbacks and other adversity will also produce differences in adaptability, thus affecting physical and mental development. Objectives: This study aimed to measure the effect of children’s emotional ability on resilience and to provide an in-depth analysis based on age and gender differences. Methodology: A total of 300 preschool children aged 3–6 years old in kindergartens of China were randomly selected as the research subjects. Through a combination of experiments and questionnaires, the emotional ability and resilience of children were measured, and differences were analyzed according to the actual situation, using age and gender. Results: Children of different ages have significant differences in the dimensions and total scores of emotional ability and resilience, but only some of the resilience dimensions have significant gender differences. Moreover, the emotional ability has a significant positive effect on resilience. Discussions: The results confirm the influence of children’s emotional ability on resilience, but the research hypothesis has not been fully verified. Limitations: This study has the limitations of a single measurement method and a more effective research tool.

## 1. Introduction

With the gradual deepening of society and continuous contact with the environment, children’s physical and mental development problems gradually increased [1,2,3]. In the health field of China’s “Learning and Development Guide for Children Aged 3–6”, it is suggested that parents and teachers help children learn to express and adjust their emotions correctly, and give appropriate guidance so that children can express their emotions [4]. Emotion is the internal development of a person’s whole life and plays an important role in personal growth [5], which will not only produce different emotions when getting along with their families, peers, and teachers [6] but also need to learn to express and adjust their emotions and understand the others’ emotions in adverse circumstances [7,8]. However, people’s performance in the face of disadvantage varies greatly [9]: some people choose to face setbacks bravely, but some people feel frustrated. The internal mechanism of this difference has attracted extensive attention from researchers. The proposal of resilience explains the appearance of this mechanism [10]. For decades, the elasticity of subjects with different ages, sexes, and backgrounds has been continuously studied by researchers [11,12]. However, there is still a lack of research on the development of children’s resilience [13]. As one of the internal protection factors of resilience, emotional ability is closely related to resilience [14]. Positive emotions can help individuals to face and adapt to adverse conditions bravely, rather than depression [9]. It is of great significance to explore the development of emotional ability and resilience in the preschool stage to cultivate children’s emotional ability and improve children’s resilience [15]. In addition, studying the influence of children’s emotional ability on resilience can provide a new way to improve the development level of children’s mental health [16].

Therefore, this study adopted a variety of research methods to explore the current status of children’s emotional ability and resilience in the context of age and gender differences. At the same time, this study also explored the influence of emotional ability on resilience. The proposal of this study would open up new perspective for the development of emotional ability and provide a new means to promote the development of resilience.

## 2. Literature Review

### 2.1. Emotional Ability 

The emotional ability was first proposed by Saarni (1990), referring to the existence of emotional ability when an individual can identify and adapt to an emotion-induced event that requires completion [17,18], which is an acquired ability based on emotional intelligence, and individuals can master and use this ability through learning [18,19]. As for the influencing factors of children’s emotional ability, it requires children to understand, express, and regulate their emotions, and experience the emotions of others [20], which would be affected by individual factors, such as age, gender, temperament, and behavior [21]; environmental factors, such as parents, peers, teachers, and cultural background [22]; and cognitive factors, such as language and music ability development [23]. Therefore, this study defined children’s emotional ability by integrating Saarni’s (1999) [18], Yao’s (2004) [24], and Li’s (2007) [25] definitions of emotional ability: children’s emotional ability consists of emotional understanding and emotional regulation; with the accumulation of age cognition and the determination of gender cognition, children have an adaptive emotional response to their abilities and social forces through emotional understanding and emotional regulation. At the same time, the development of children’s emotional understanding and emotion regulation strategies as an important part of emotional ability development can help us better understand the development of children’s emotional ability [26].

#### 2.1.1. Emotional Understanding

Children’s emotional understanding has been widely concerned and studied [27]. Pons et al. (2004) proposed that emotional understanding includes emotional recognition, context attribution, desire, belief, emotional suggestion, emotional hidden, mixed emotion understanding, and moral emotion understanding [28]. According to the characteristics of children’s physical and mental development, this study selected expression recognition, emotional viewpoint selection, emotional attribution interpretation, belief-based emotional understanding, desire-based emotional understanding, and conflict emotional understanding for the evaluation of children’s emotional understanding.

#### 2.1.2. Emotional Regulation

Emotional regulation is the core component of children’s social-emotional development and plays an important role in children’s emotional ability. Parkinson’s and Totterdell (1999) used a questionnaire to analyze and classify emotional regulation into two types, one being cognitive and behavioral strategies, the other being attention and transfer strategies [29]. Eisenberg (1994) based on the three dimensions of attentional regulation, emotional expression, and behavioral regulation proposed by emotional regulation, and studied the emotional regulation ability of children with experimental methods [30]. In this study, according to the characteristics of children, the problem-based approach was used to study the emotional regulation of children. Due to the children’s age limit, the parents of the selected children filled in the children’s emotional adjustment questionnaire.

According to previous studies, it is not difficult to find that researchers pay more attention to the development of emotional ability. The overall research on children’s emotional ability is at a weak level and still needs to be improved. Therefore, it is very important to carry out an in-depth and comprehensive study on children’s emotional ability.

### 2.2. Resilience 

The concept of resilience is different due to research in different periods, backgrounds, and objects [31,32,33], which is mainly defined from three aspects: result [34], process [35], and ability [36]. Moreover, Ungar (2011) mentioned that resilience exists generally in individuals, but the difference in the development level of resilience is caused by different physical and mental development. Therefore, it can be improved through postnatal training [37] and individuals can improve their resilience through learning and use them well in future crises [37,38]. To sum up, this study adopted Masten’s (2014) definition [39] to define resilience as a kind of dynamic ability and considered that children’s resilience is the adaptability and recovery of children in the face of difficulties, setbacks, and conflicts, and there are significant individual dynamic changes with different cultures, personal encounters, family, gender, age, and so on. 

According to different research objects and factors affecting resilience, researchers are more inclined to use scales to study resilience. Therefore, many different resilience scales have been proposed in the existing studies and have been widely used in previous studies [40]. Example include the Brief Resilience Coping Scale [41], the Devereux Early Childhood Assessment [42], and the Devereux Early Childhood Assessment (DECA) Scale Second Edition [43]. According to the actual physical and mental development of Chinese children, Liang et al. (2019) conducted a Chinese reliability and validity analysis of the Devereux Early Childhood Resilience Assessment Scale Second Edition [44]. Mandelco (2000) pointed out that resilience includes internal factors composed of biological and psychological factors, and external factors composed of the environment [45]. Ma et al. (2018) divided the indicators that reflect the resilience of young children into three aspects: individual factors, family factors, and extra-family factors [46,47]. Zhang (2019) divided the factors that affect young children’s resilience into two aspects: internal factors based on themselves and external factors based on the environment [48]. Internal factors include gender, physical fitness, curiosity and interest, self-confidence, self-expectation, security, and communication skills. External factors include family education, interpersonal relationships, teacher factors, and the consistency of home education [45]. To sum up, the resilience of young children is affected by internal factors and external factors [49]. Therefore, in order to study resilience with the experimental method, this study needs to restore the relevant characteristics of resilience to the present experiment. Firstly, create an unfavorable experimental situation for the subjects. Secondly, observe the cognitive behavior performance of the subjects in the experimental situation. 

In summary, although previous studies have involved related research about resilience on internal and external factors, researchers rarely the resilience of young children. Therefore, the research on the resilience of children is of great significance.

### 2.3. The Relationship between Emotional Ability and Resilience

As one of the protection factors of resilience, emotional ability is closely related to resilience [50]. Roach (2016) showed that the positive emotional level of individuals with higher elasticity was higher than the emotional level of individuals with lower elasticity [51]. Ainize (2018) also pointed out that the improvement of elasticity could reduce the connection between stress and negative emotions [52]. Rees (2013) pointed out that there is an obvious positive correlation between emotional literacy and resilience on children’s mental health [53]. On the physiological level, Motsan et al. (2021) discussed the interaction of biological, emotional, cognitive, and relational factors to form two kinds of regulatory results in young people exposed to trauma: emotional recognition and executive function. The results showed that early traumatized children had certain barriers to emotion and resilience, which led to different clinical manifestations in physiology [54]. To sum up, emotion and resilience are factors cannot be ignored in the process of children’s growth, which may affect future physical and psychological development. Therefore, it is of great significance to explore the relationship between emotional ability and resilience.

### 2.4. Age and Gender as Different Roles

At the same time, in-depth analysis is needed to deeply grasp the role of gender and age as different roles between emotional ability and resilience. Through combing the previous literature, studies on the emotional ability and resilience of young children have confirmed that they are related to age and gender, but they failed to identify the differences arising from the relationship between the two [50,51,55,56]. Zeeman et al. (2017) confirmed that the performance of emotional ability and resilience in young groups are affected by gender and sexual orientation [57]. Lee and Moon (2017) pointed out that school-age children have differences in emotional regulation and resilience [58].

Researchers believed that age is the decisive factor of emotional ability and resilience, and found that both will increase with age [18,24,25]. As mentioned above, the differences brought by the age of individuals as life cycles will be most easily observed, but whether they also exist in this study needs further verification. Gender differences have not been widely mentioned in previous studies on emotional ability and resilience. However, as mentioned above, gender will have different reactions when facing different situations in early childhood, so the gender differences mentioned in a few articles cannot be ignored [39]. At the same time, Sun (2007) pointed out in the research on children’s resilience in Brisbane that the cross-action of age and gender is very important for resilience, which inspired this study to study the differences between age and gender, but at the same time, we should not ignore their cross-action [59].

Drawing on these findings, we note that age and gender are objective factors that cannot be ignored when discussing the relationship between emotional ability and resilience [58,59]. However, the research on children still needs to be explored and improved. The trauma in early childhood will affect the shaping and development of their personality in their whole life. Improving children’s psychological elasticity can reduce the influence of external factors on children [60]. Therefore, this study investigated children’s emotional ability and resilience; it then explored the influence of emotional ability on children’s resilience, as well as the differences brought by age and gender.

## 3. Materials and Methods

### 3.1. Participants

In this study, 300 children aged 3–6 years were selected from Jiangsu Province and Fujian Province by simple random sampling, of which 150 were from a kindergarten in Jiangsu Province and 150 were from a kindergarten in Fujian Province. The reason for choosing these two kindergartens is that the kindergarten environment, teacher matching, and the number of students are almost the same, which can reduce the influence of other factors. First of all, the selection of all participants was confirmed by random lottery to ensure that the selection probability of each participant was equal. Secondly, Jiangsu Province is a relatively developed region in China, while Fujian Province is a region with a medium development level. The main reason for choosing these two places was to control the influence of the differences in the development level of research fields. In the final analysis of this study, it was also found that the difference between the two regions is not significant, so the final data can be combined and analyzed. Third, all participants needed the consent of their guardians and could participate anonymously. Finally, 5 participants who were unable to participate in the experiment due to illness, absence of parents, or other reasons were excluded. In addition, the final data-screening process also included responses to 6 missing data. Finally, 289 valid data were obtained. See Table 1 below for details. 

### 3.2. Methods

#### 3.2.1. Emotional Ability

Emotional ability is divided into two parts: emotional understanding and emotional regulation. Emotional understanding was assessed by teachers, using classroom experiments, while emotional regulation was assessed by parents’ assessment of children’s performance in the context. The specific operations are as follows.

##### Emotional Understanding Experiment

According to previous research and the intelligence development of children, the measurement of emotional understanding is divided into the following six experiments in this study: expression recognition [61], perspective taking, emotional attribution [62], belief understanding, desire understanding, and conflict understanding [63] (see Appendix A for detailed experimental operation). The reason why the scale is not used is to reduce the inaccuracy of the data caused by the incomprehension of children’s word cognition level. 

At the same time, to ensure the accuracy of the data in the emotional understanding part, the Pearson correlation analysis was used to verify the results of the six group experiments, which showed that there was a significant positive correlation between each dimension of emotion understanding (*p* < 0.001). The results are shown in Table 2. The coefficient of the emotional understanding scale was 0.771.

##### Emotional Regulation Scale

**Figure A4:**
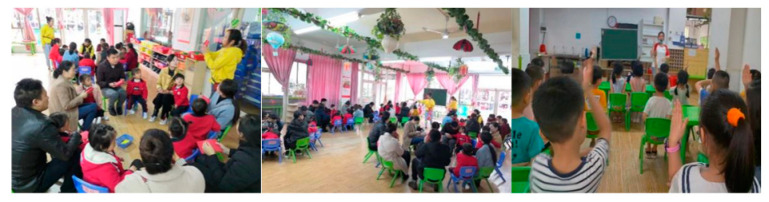
Parents participate in activities to observe children’s performance.

The investigation of young children’s emotional regulation adopts the Questionnaire of Young Children’s Emotional Regulation Strategies [64] used by Callear (2018). After being processed by the subjects, the questionnaire retained a total of 17 items (see Appendix B Table A2). Positive emotional regulation includes five dimensions: cognitive reconstruction, problem-solving, seeking support, alternative activities, and self-comfort. Negative emotional regulation includes three dimensions: passive coping, emotional venting, and aggressive behavior. The scale uses a five-level rating and completed by parents (see Appendix A Figure A4). The coefficient of the emotional regulation scale was 0.834.

##### Calculation of Emotional Ability

Tian (2016) [65] pointed out that the evaluation of emotional ability consists of emotional understanding and emotional regulation. Therefore, the analysis of emotional ability is to form complete data by integrating the data of emotional ability and emotional regulation, which requires re-analyzing whether its reliability and validity can be applied to this study. Due to the different assignment methods of emotional understanding and emotional regulation, it is necessary to reprocess the data before merging. Kolen and Brennan (2004) pointed out that the linear equivalent method can deal with the problem of different scores between scales, so that it can explain the results of the two scales meaningfully [66]. In addition, it is considered that all variables have the same weight, so no extra weight calculation is needed. After analysis, the coefficient of the emotional ability scale was 0.877, which can be used to evaluate the resilience of children.

#### 3.2.2. Resilience Scale

Children’s resilience was assessed by the Chinese Version of the DECA revised by Liang (2019) [44]. After being processed by the subjects, the questionnaire retained a total of 10 items (see Appendix B Table A3), which were divided into four dimensions: initiative, self-regulation, attachment/relationship, and behavior problems. It should be reminded that self-regulation in resilience should be different from emotional regulation, which refers to behaviors made in the situation, not the emotion expressed [44]. The scores of initiative, self-regulation, and attachment/relationship are converted into T scores and added together to form the overall protective factor score. The overall protective factor is used as the standard to measure the development level of children’s mental resilience. The dimension of behavioral problems is not an overall protective factor and serves as a screening mechanism for children’s behavioral problems. The scale uses a five-level rating and completed by parents (see Appendix A Figure A4). The coefficient of resilience scale was 0.901.

### 3.3. Procedure

There were two experiments in this study. The first experiment (emotional understanding) was conducted by the kindergarten teachers in the classroom and included six experiments. Teachers filled in the emotional understanding record form according to the standard of children’s performance score in this study (see Appendix B Table A1). The purpose of this experiment was to understand the current situation of children’s emotional ability. The second experiment was carried out under the cooperation of kindergarten and parents. First, it was to create various possible situations for the children. Second, it was to observe the children’s response to these processes, thus producing emotions, behaviors, and performances, as well as self-regulation. Finally, the parents filled out the children’s emotional regulation and resilience scale (see Appendix B Table A2 and Table A3 ). The purpose of this experiment was to understand the development status of children’s resilience.

In addition, after obtaining the data from two experiments, this study carried out a formal analysis process on the data and put forward research hypotheses. According to the needs of the research hypotheses, through descriptive statistics, difference analysis, and correlation analysis, they can figure out the current situation, difference, and the relationship between children’s emotional ability and resilience.

### 3.4. Research Hypothesis

Based on the literature reviews, the following research hypotheses were proposed:

**Hypothesis** **1.** *According to the literature review, age, gender, and their cross-effects have a significant impact on children’s emotional ability and resilience. Therefore, based on the above judgment, this study puts forward the research hypothesis: there are significant differences in emotional ability and children’s resilience at different age and gender levels.*

**Hypothesis** **2.** *According to the literature review, children’s emotional ability consists of two parts: emotional understanding and emotional regulation. At the same time, previous studies have shown that emotional ability is one of the important factors affecting resilience. Therefore, based on the above judgment, this study puts forward the research hypothesis: children’s emotional ability is positively correlated with children’s resilience.*

**Figure 1:**
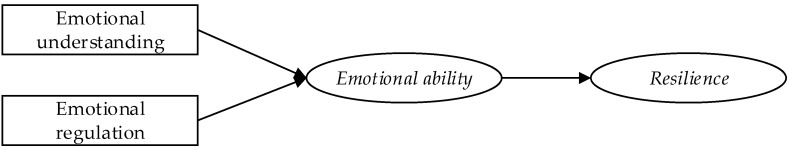
Hypothesis model.

**Hypothesis** **3.** *There is a hypothesis model (Figure 1) that can explain the path effect between emotional ability and resilience.*

### 3.5. Data Analysis

In order to determine whether the measurement has satisfactory psychometric attributes, SPSS 25.0 software was used to analyze the scale, and the reliability of the subscale was evaluated by Cronbach α coefficient. Secondly, descriptive statistics were used to carry out the quantitative distribution of age and sex, and judge whether the sample distribution is suitable for the next analysis. Thirdly, the differences of age, sex, and age x sex were analyzed; the differences of emotion understanding, emotion regulation, and resilience were obtained; and the differences brought by each age and sex were obtained to verify the research Hypothesis 1. Fourthly, we used a single linear regression to analyze the relationship between emotional ability and resilience, so as to verify research Hypothesis 2. At last, structural equation modeling (SEM) via Amos 24.0 was used to construct a full model to explore the relationships among emotional understanding, emotional regulation, emotional ability, and resilience, in order to ensure the verification of research Hypothesis 3.

## 4. Results

### 4.1. Differences Analysis Based on Age and Gender

#### 4.1.1. Emotional Understanding, Age, and Gender

Firstly, all dimensions of emotional understanding were described according to age and gender. Then, this study took age and gender as independent variables and each dimension as dependent variables and conducted a multivariate analysis of variance to investigate the differences in age and gender. The specific results are shown in Table 3. As far as age is concerned, the dimension of children’s emotional understanding increases with age. Moreover, in regard to gender, girls are higher than boys in expression recognition, emotional attribution, and conflict understanding. In perspective-taking, belief understanding, and desire understanding, boys are higher than girls.

In order to explore the differences brought by age and gender, this study chose to conduct the main effect and interaction effect analysis. The results showed the following: Firstly, there was a significant difference in age in all dimensions of emotional understanding. Secondly, in terms of gender, there is no significant difference in the main gender effects of each dimension of emotional understanding. Thirdly, under the interaction of age and gender, there is no significant difference in each dimension of emotional understanding. As the above results indicated that there existed age differences, in order to further analyze the differences caused by age, this study chose to conduct analysis through one-way ANOVA (LSD) of age, which shows all dimensions of emotional understanding are basically getting better with age, as well as the total score of emotional understanding.

#### 4.1.2. Emotional Regulation, Age, and Gender

Firstly, this study also takes age and gender as independent variables and each dimension as dependent variables, and conducts a multivariate analysis of variance to investigate the differences in age and gender. The specific results are shown in Table 4. As far as age is concerned, children’s emotional regulation generally increases with age, but not all dimensions. Moreover, in regard to gender, the development level of girls is higher than that of boys in cognitive reconstruction, problem-solving, self-comfort, seeking support, and passive coping. The development level of boys is higher than that of girls in alternative activities, emotional venting, and aggressive behaviors. In terms of the total score of emotional regulation, the total score of boys’ emotional regulation was 18.79 ± 2.531, while that of girls was 18.90 ± 2.323, and the development level of girls was higher than that of boys.

In the analysis of the main effect and interaction effect, first of all, the main effect of cognitive reconstruction, problem-solving, self-comfort, passive coping, emotional venting, and the total of emotional regulation on age was significant. Secondly, in the main effect of gender, aggressive behavior has a significant gender difference. Finally, under the interaction of age and gender, there is no significant difference in all dimensions of children’s emotional regulation. As the above results indicated that there existed age differences, in order to further analyze the differences caused by age, this study chose to conduct analysis through one-way ANOVA (LSD) of age. The results showed the following: in cognitive reconstruction, 6 > 3, 4; in problem-solving, 6 > 3, 4, 5; 5 > 3; in self-comfort, 6 > 3, 4, 5; in passive coping, 6 > 3, 4, 5; 5 > 3; and in emotional venting, 3, 4 > 6; 3, 4 > 5. In the total score of emotional regulation, there were significant differences between 3-year-old and 6-year-old children, and between 4-year-old and 6-year-old children.

#### 4.1.3. Resilience, Age, and Gender

According to the exploration of the Devereux Early Childhood Assessment, the level of children’s resilience can be divided into three types: those with a score of 40 or below indicate that children’s resilience level is low and needs to be improved through external guidance; a score of 40–60 points indicates that children’s resilience level is general; and a score above 60 points indicates that children’s resilience level is higher. In term of the score of behavior problems, the development of children’s behavior problems is divided into two stages: those with a score below 60 indicate that children’s behavior problems are at a normal level; those with a score above 60 indicate that children’s behavior problems are higher than normal level, which should be paid attention to by parents and teachers. 

In this study, the three dimensions have the same distribution in the three levels of resilience. From the overall protective factors, it can be seen that, in this study, 16% of children’s resilience level is low, 68% of the children are in the general level, and 16% of the children are higher. In the dimension of problem behavior, 84% of children’s behavior problems are at a normal level, and 16% of children’s behavior problems are higher than the normal level. The specific results are shown in Table 5.

As far as age is concerned, the initiative, self-regulation, and total of children’s resilience showed certain development in age. While in gender, girls’ initiative, self-regulation, attachment/relationship, and overall protective factors are higher than boys, the behavior problems of boys were higher than those of girls.

In the analysis of the main effect and interaction effect, first of all, there was a significant difference in the main effect of age on the initiative, self-regulation, and attachment/relationship. Secondly, the initiative, attachment/relationship, and the total score of resilience had a significant difference in gender main effect. Finally, under the interaction effect of age and gender, the total scores of resilience were significantly different. As the above results indicated that there existed age differences, in order to further analyze the differences caused by age, this study chose to conduct analysis through one-way ANOVA (LSD) of age. The results showed the following: in initiative, 5, 6 > 3; in self-regulation, 5, 6 > 3; 5, 6 > 4; and in attachment/relationship, 3 > 6; 4 > 6.

#### 4.1.4. Age and Gender Differences in Emotional Ability and Resilience

**Figure 2:**
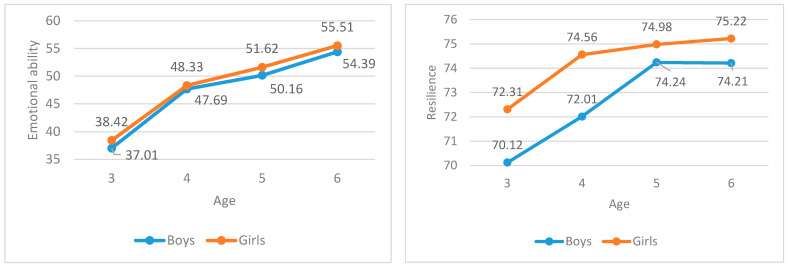
Age and gender differences in emotional ability and resilience.

In this study, the score of emotional ability was obtained after the score of emotional understanding and emotional regulation is standardized and assigned. Moreover, the differences of different ages and genders were statistically analyzed, and the results shown in Figure 2 below are obtained.

It can be seen from Figure 2 that, with the increase of age, the emotional ability gradually increases, and the overall level of girls is slightly higher than that of boys at all ages. This further verifies the results obtained in Section 4.1.1 and Section 4.1.2. Moreover, with the increase of age, the resilience also gradually increases, and the overall level of girls is higher than that of boys at all ages. This trend is generally consistent with emotional ability. However, it can be seen from Figure 2 that the resilience of boys aged 3 and 4 is significantly lower than that of girls, but the gap is gradually shortened after the ages of 5 and 6. This further verifies the results obtained in Section 4.1.3.

### 4.2. Correlation Analysis of Emotional Ability and Resilience

As can be seen from Table 6, emotional understanding, emotional regulation, and emotional ability are significantly related in several dimensions of resilience, except attachment/relationship. This shows that the four variables meet the standard requirements of correlation analysis, so the next step of regression analysis can be carried out.

### 4.3. Regression Analysis of Emotional Ability and Resilience

It can be seen from Table 7 that the regression analysis is carried out with children’s emotional understanding, emotional regulation, and emotional ability as independent variables and resilience as the dependent variable. The results show that there are significant linear relationships between children’s emotional understanding, emotional regulation, emotional ability, and resilience, and a linear regression equation can be established.

The regression equation is as follows:

(1) Resilience=133.787+0.402∗Emotional understanding

(2) Resilience=103.242+2.154∗Emotional regulation

(3) Resilience=121.969+0.537∗Emotional ability

### 4.4. Path Effect Analysis of Emotional Ability and Resilience

The results show that there is significant positive correlation between emotional understanding and emotional regulation on emotional ability, and the relationship between emotional ability and resilience. Therefore, this study verified the model of Hypothesis 3. 

In this study, Amos 22 was used to test the model and the goodness of fit index was obtained as shown in Table 8. The results show that the hypothesis model is acceptable. 

Because emotional ability is composed of emotional understanding and emotional regulation, it should be included both of them while analyzing emotional ability and resilience. Therefore, this study used SPSS to test path effect of emotional ability and resilience, as well as emotional understanding and emotional regulation. The results in Table 9 showed the mediator of emotional ability and resilience is significant.

**Figure 3:**
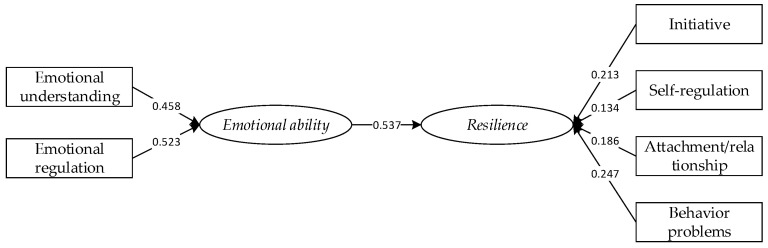
Hypothesis model.

Therefore, Hypothesis 3 is validated, and the model results are shown in Figure 3.

## 5. Discussion

### 5.1. The Research of Children’s Emotional Ability, Age, and Gender

Firstly, in the emotional understanding part. The hypothesis is that gender and age will cause differences in emotional understanding, but the results showed that only age has a difference, and gender has no significant difference in emotional understanding. Based on the previous literature review, Zhang (1990) showed that children do not have complete gender awareness until they are in fifth grade. From kindergarten to the fifth grade, boys show a consistent preference for male objects and occupations, while girls show no gender preference at kindergarten age [67]. Therefore, 3–6-year-old children may not have significant differences in emotional understanding due to different gender perceptions [67].

Secondly, in the emotional regulation part. The hypothesis is that gender and age will cause differences in emotional regulation. The result showed that children’s positive emotions increased with age and there are significant differences in cognitive reconstruction, problem-solving, self-comfort, passive coping, emotional venting, and the total score of emotional regulation. At the same time, there are significant gender differences in aggressive behavior. Boys are more negative than girls in emotional regulation. However, there was no significant interaction between age and gender. Moreover, this study did not find that gender can cause significant differences in the aggressive behavior of children, so the results are worthy of attention. Future research should focus on why children have gender differences in aggressive behavior. Zhang et al. (2015) pointed out that the generation of aggressive behavior may be more related to the relationship and family parents [68]. 

### 5.2. The Research of Children’s Resilience, Age, and Gender

Firstly, there are significant differences in the dimensions of initiative, self-regulation, and attachment/relationship in age. At the same time, initiative and self-regulation increased with age, while attachment/relationship decreased. This result is consistent with that of Zhang (2017). With the growth of children’s age and mental maturity, the degree of dependence on parents will gradually decrease [67]. The formation of personal domain consciousness helps children constantly form active consciousness and form their own unique emotional regulation form. However, there is no significant difference between cognition and self-regulation. As Zhang (1990) said, the cognitive level of 3–6-year-old children will continue to grow with age, but the difference is not significant, and they are in the embryonic stage of development [68]. 

Secondly, there are significant gender differences in the initiative, attachment/relationship, and overall protective factors. Generally speaking, the resilience level of girls is higher than that of boys, and the behavioral problems of boys are more than that of girls. This result is consistent with the findings of Xu (2018); that is, in terms of social adaptation, girls have stronger social adaptability and have fewer problem behaviors, while boys have poor social skills and are prone to conflict and have more behavioral problems [69].

In conclusion, the results of Section 5.1 and Section 5.2 showed that age has significant difference in emotional ability and resilience, but gender has no significant difference in emotional ability and resilience. This study verified the age difference in Hypothesis 1, but not gender.

### 5.3. The Relationship between Children’s Emotional Ability and Resilience

Firstly, the higher emotional understanding, emotional regulation, and emotional ability, the higher resilience. The higher children’s positive emotional regulation is, the higher children’s resilience is. The results of this study confirmed that, like adults, children’s emotional ability has an impact on resilience. Even if children are younger and less involved in social factors, the higher emotional ability has a significant effect on children’s resilience. Moreover, this study also made up for the findings of the previous studies, the lack of children as the research participants. At the same time, this study also confirmed it is better to pay attention to the improvement of children’s emotional ability than to the development of children’s emotional intelligence. After the emotional ability is guaranteed, other factors of emotional intelligence, such as teamwork and self-efficacy, can be further improved. That is only in the premise of self-assurance, children can better explore communication with others and environmental exploration.

Secondly, there is a significant positive correlation between emotional ability and resilience. In this study, the relationship between emotional ability and resilience was analyzed by regression analysis. Taking emotional ability as an independent variable and resilience as a dependent variable, a regression equation was obtained. The results found that emotional ability has a significant positive effect on resilience. At the same time, it verifies the results of previous studies, with the growth of age, the emotional ability will increase, which has an important positive effect on resilience. This verifies Hypothesis 2.

Finally, emotional understanding and emotional regulation have significant effects on emotional ability, which helps emotional ability to have an effect on resilience. This result showed that the contribution of emotional understanding to emotional ability is 0.458, while that of emotional regulation is 0.523, which proves that emotional understanding and emotional regulation are the two main components of emotional ability. Therefore, more attention should be paid to the formation of children’s ability in these two aspects. At the same time, the contribution of emotional ability to resilience reached 0.537, which also confirmed that emotional ability cannot be ignored in children’s resilience, and provided a new research dimension for the selection of influencing factors of children’s resilience in future research. This verifies the Hypothesis 3.

### 5.4. The Gender Difference

Gender as one of the variables did not yield effective results. There is undoubtedly an agreement that it is essential to consider children’s cultural context if one is to understand their cognitive development [70]. At the same time, Brody (1999) proposed that girls’ self-control ability is higher than boys’ in early childhood due to genetic and hormonal differences [71]. Considering Chinese local cultural background, most children spend more time with their mothers. When a girl is wrong or disobedient, her mother can give her a patient explanation and guidance. The girl may better control emotions to solve the problem [72]. This may also explain why the overall level of girls in this study was slightly higher than boys. However, as children’s self-control ability continues to improve during childhood [73], both boys and girls in the first grade (aged 6–7) of primary school may be influenced by maternal nonviolent discipline to control negative emotions rationally. This can also explain the situation that the level of emotional ability and resilience of children aged 5–6 years old in this study is gradually stable compared with the level of children aged 3–4 years old, which has significantly increased every year. Hence, although this study takes gender as one of the variables, it does not reflect this gender difference.

## 6. Limitations

This study took 3–6-year-old children as research participants to explore the relationship between emotional ability and resilience. Through the experiment and questionnaire survey, some results were obtained, but there were still some deficiencies in the details.

Firstly, there were deficiencies in the sample selection and research of the hypotheses. In this study, there was no uniform age and gender distribution in the choice of subjects, which led to the deviation between the research results and previous studies. In addition, this study took children aged 3–6 years in some provinces of China as research objects, which may have led to the ideal results of this study. Therefore, this study shows that future research can focus on left-behind children in backward areas or children from single-parent families in backward areas, and these studies may have more different conclusions.

Secondly, lacking effective research methods, this study only used experimental methods to study children’s emotional understanding. In the research of emotional regulation and resilience, parents were investigated by questionnaire. Parents’ subjective initiative may lead to deviation in the results. Therefore, future research can also try to develop a table of emotional ability and resilience suitable for children’s testing, and conduct more targeted experiments to ensure that the credibility of the experiment is not affected by the subjectivity of others.

Finally, the research tools were single. This study explored the development level of children’s resilience through the children’s resilience assessment scale. Only initiative, self-regulation, attachment/relationship development, and so on are slightly one-sided, so the future study should pay more attention to other factors affecting children’s resilience development. Therefore, this paper thinks that future research can also try to do some qualitative research or field research and carry out long-term tracking to obtain data. The combination of these two experimental methods will help to analyze the influencing factors of children’s emotional ability and resilience more comprehensively.

## Figures and Tables

**Table 1:** General information.

Age	Male	Female	Percentage
3 years old	32	26	20.07%
4 years old	45	39	29.07%
5 years old	38	42	27.68%
6 years old	32	35	23.18%
Total	147	142	100%

**Table 2:** Correlation analysis of emotional understanding.

Variable	1	2	3	4	5	6	7
1. Expression recognition	-						
2. Perspective taking	0.558 ***	-					
3. Emotional attribution	0.424 ***	0.705 ***	-				
4. Belief understanding	0.378 ***	0.385 ***	0.338 ***	-			
5. Desire understanding	0.411 ***	0.488 ***	0.482 ***	0.526 ***	-		
6. Conflict understanding	0.407 ***	0.549 ***	0.477 ***	0.348 ***	0.426 ***	-	
7. Emotion understanding	0.718 ***	0.878 ***	0.846 ***	0.567 ***	0.668 ***	0.652 ***	-

Note: *** *p* < 0.001.

**Table 3:** General information and difference analysis of emotional understanding.

		Expression Recognition		Perspective-Taking		Emotional Attribution		Belief Understanding		Desire Understanding		Conflict Understanding		The Total Score of Emotional Understanding	
		M	SD	M	SD	M	SD	M	SD	M	SD	M	SD	M	SD
Age	3	11.90	1.555	3.00	1.796	3.04	2.730	0.40	0.563	0.22	0.459	0.64	0.608	19.20	5.711
	4	13.88	1.261	6.08	1.626	6.53	1.775	0.81	0.778	0.89	0.768	1.29	0.633	29.48	4.841
	5	14.47	1.071	6.84	1.443	6.72	1.678	1.25	0.829	1.28	0.723	1.52	0.556	32.08	5.301
	6	15.09	0.930	7.58	0.622	7.45	0.747	1.71	0.578	1.75	0.400	1.75	0.315	35.33	2.592
	**F**	66.818 ***		115.678 ***		56.877 ***		35.662 ***		47.605 ***		32.752 ***		169.441 ***	
Sex	Boys	13.78	1.625	5.90	2.255	5.76	2.602	1.07	0.824	1.04	0.809	1.24	0.681	28.79	6.796
	Girls	13.81	1.690	5.76	2.223	6.08	2.299	0.94	0.853	0.95	0.786	1.32	0.645	28.86	6.496
	**F**	0.12		0.12		1.634		1.386		0.524		1.612		0.258	
Age × Sex	**F**	0.392		1.012		0.209		0.632		0.235		0.21		0.268	
Post analysis		6 > 5 > 4 > 3		6 > 5 > 4 > 3		6, 5, 4 > 3; 6 > 4		6 > 5 > 4 > 3		6 > 5 > 4 > 3		6, 5, 4 > 3; 5, 6 > 4		6 > 5 > 4 > 3	

Note: F means variance test; *** *p* < 0.001.

**Table 4:** General information and difference analysis of emotional regulation.

		Cognitive Reconstruction		Problem- Solving		Self- Comfort		Alternative Activities		Seek Support		Passive Coping		Emotional Venting		Aggressive Behavior		The Total Score of Emotional Regulation	
		M	SD	M	SD	M	SD	M	SD	M	SD	M	SD	M	SD	M	SD	M	SD
Age	3	2.53	0.484	2.56	0.548	1.85	0.604	2.91	0.437	2.94	0.661	2.07	0.559	1.88	0.555	1.69	0.577	18.43	2.371
	4	2.61	0.568	2.63	0.438	1.81	0.556	2.95	0.520	3.07	0.571	2.21	0.522	1.81	0.642	1.56	0.491	18.56	2.151
	5	2.73	0.563	2.74	0.535	1.84	0.644	3.02	0.521	3.00	0.653	2.25	0.580	1.63	0.549	1.59	0.615	18.80	2.760
	6	2.88	0.587	3.02	0.542	2.20	0.716	3.13	0.479	2.97	0.573	2.49	0.458	1.52	0.333	1.45	0.398	19.66	2.262
	Total	2.68	0.543	2.72	0.539	1.92	0.638	3.02	0.497	3.00	0.615	2.25	0.550	1.71	0.561	1.58	0.537	18.88	2.427
	**F**	3.221 *		8.784 ***		4.522 **		2.018		0.606		5.979 **		5.508 **		2.2989		2.697 *	
Sex	Boys	2.64	0.586	2.73	0.552	1.88	0.678	3.00	0.483	2.95	0.591	2.22	0.542	1.73	0.510	1.64	0.514	18.79	2.531
	Girls	2.70	0.514	2.75	0.532	1.93	0.598	2.99	0.512	3.07	0.635	2.26	0.560	1.71	0.608	1.49	0.549	18.90	2.323
	**F**	0.264		0.232		0.172		0.045		2.849		0.079		0.279		4.766 *		0.039	
Age × Sex	**F**	0.878		0.38		0.91		0.071		0.769		1.157		0.448		0.061		0.758	
Post analysis		6 > 3, 4		6 > 3, 4, 5; 5 > 3		6 > 3, 4, 5						6 > 3, 4, 5; 5 > 3		3, 4 > 6; 3, 4 > 5				6 > 3, 4	

Note: F means variance test; * *p* < 0.05; ** *p* < 0.01; *** *p* < 0.001.

**Table 5:** General information and difference analysis of resilience.

		Initiative		Self-Regulation		Attachment/Relationship		Behavioral Problems		The Total Score of Resilience	
		M	SD	M	SD	M	SD	M	SD	M	SD
Age	3	23.44	3.578	15.52	2.945	19.49	2.534	13.12	4.898	71.57	6.989
	4	24.33	3.646	16.08	2.096	19.68	2.354	13.01	4.678	73.11	6.112
	5	25.48	4.358	17.01	2.899	19.17	2.467	13.01	5.087	74.67	5.977
	6	25.68	4.397	17.49	2.787	18.37	3.366	13.07	3.822	74.61	6.696
	**F**	4.192 **		6.165 ***		2.766 *		0.039		2.002	
Sex	Boys	24.08	4.080	16.28	2.834	18.87	2.855	13.54	4.888	72.77	6.333
	Girls	25.32	3.898	16.67	2.729	19.67	2.338	12.45	4.345	74.11	5.459
	**F**	8.678 **		1.916		6.878 **		3.262		9.787 **	
Age × Sex	**F**	3.345 *		1.198		1.787		0.087		3.399 *	
Post analysis		5, 6 > 3		5, 6 > 3; 5, 6 > 4		3 > 6; 4 > 6					

Note: F means variance test; * *p* < 0.05; ** *p* < 0.01; *** *p* < 0.001.

**Table 6:** Correlation analysis of emotional ability and resilience.

	Initiative	Self- Regulation	Attachment/ Relationship	Behavioral Problems	The Total Score of Resilience
Emotional understanding	0.157 **	0.198 **	−0.074	−0.051 **	0.123 *
Emotional regulation	0.277 ***	0.278 ***	0.011	0.022 ***	0.258 ***
Emotional ability	0.233 ***	0.273 ***	−0.064	−0.117 ***	0.199 ***

Note: * *p* < 0.05; ** *p* < 0.01; *** *p* < 0.001.

**Table 7:** Regression analysis of emotional ability and resilience.

Model	Variables	B	t	R	R^2^	∆R^2^	F
1	Constant	133.787	23.122 ***	0.121	0.017	0.014	4.857 *
	Emotional understanding	0.402	2.123 *				
2	Constant	103.242	9.898 ***	0.257	0.069	0.067	19.899 ***
	Emotional regulation	2.154	4.358 ***				
3	Constant	121.969	14.666 ***	0.212	0.039	0.037	11.689 *
	Emotional ability	0.537	3.389 **				

Note: * *p* < 0.05; ** *p* < 0.01; *** *p* < 0.001.

**Table 8:** Fit indices of the hypothesis model.

χ2	df	χ2/df	CFI	NFI	TLI	IFI	AGFI	RMSEA
79.901	27	2.959	0.971	0.955	0.959	0.971	0.951	0.058

**Table 9:** Direct path effect. (DE, direct effect; there is no indirect effect; the table does not show IE.)

Path	DE	BootLLCI	BootULCI
emotional understanding→emotional ability	0.458	0.409	0.789
emotional regulation→emotional ability	0.523	0.332	0.689
emotional ability→resilience	0.537	0.413	0.698

**Figure A1:**
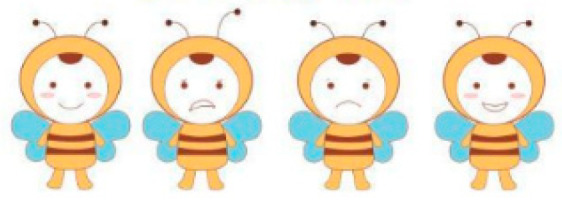
Identification map of happiness, anger, sadness, and joy.

**Figure A2:**
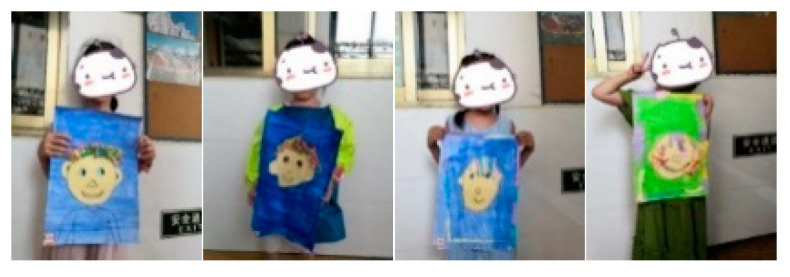
Children express their understanding of joy by drawing.

**Figure A3:**
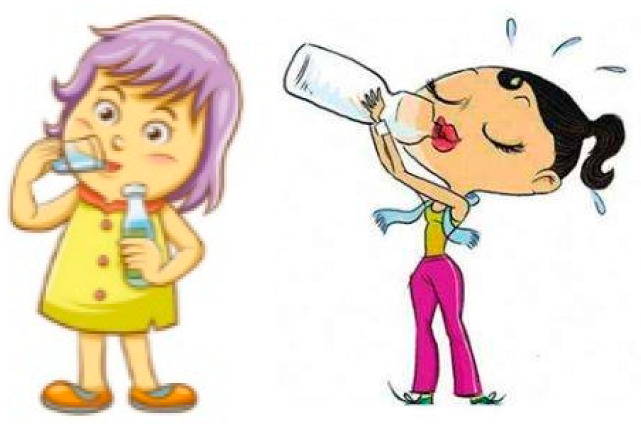
Emotion understanding—Coco drinking water.

## Data Availability

The data that support the findings of this study are available from the corresponding author. Restrictions apply to the availability of these data, which were used under licence for this study. Data are available from the authors with the permission of Nanjing Normal University.

## Appendix A

### Appendix A.1. Six Experiments of Emotional Understanding

#### Appendix A.1.1. E1: Expression Recognition Experiment

This experiment uses the experimental method adopted by Garcia (2020) to investigate the children’s understanding of facial expressions. First, use the selected facial expression pictures to test the children. The expressions in the facial expression pictures include happiness, anger, sadness, and joy (see Appendix A Figure A1). The expression recognition experiment is divided into an expression naming task and a recognition task. The expression naming task asked the children to orally confirm the facial expression pictures one by one, and the researchers asked the children: what happened to the children in the picture? The children were asked to answer the expression shown in the picture. The expression recognition task requires the children to find the corresponding facial expression pictures under the questions of the researchers or to describe the facial expressions in the form of painting (see Appendix A Figure A2). The scoring standard of the expression recognition experiment adopts a two-point scoring method, and the total score of the expression naming task and the expression recognition task is between 0 and 16 points. Participants receive 2 points for exactly correct, 1 point for being able to express the positive or negative emotional state corresponding to the expression and 0 points for irrelevant answers or incorrect answers.

#### Appendix A.1.2. E2: Emotional Viewpoint Selection Experiment

This experiment uses Liu’s (2006) Viewpoint Selection Scenario Story. The researcher provided and told the children’s situational stories to examine the children’s judgment level of the emotional state of others in the situation. When the child is unable to express his/her emotional state, he/she can provide pictures for facial expression recognition tasks for selection. Situational stories need to exclude gender and name interference. Each basic emotion is equipped with a situational story, and the researcher presents the four situational stories in random order. For example, there is a child named Coco who likes kittens very much. On his birthday, his father brought a kitten back to him. How would Coco feel? The child needs to answer happy feelings, or identify happy expressions in pictures or express positive feelings by identifying facial expressions. The scoring standard is based on a 2-point scoring method. The total score of the emotional viewpoint selection task is between 0 and 8. Completely correct emotional opinions are scored 2 points, and those who can express positive or negative emotions corresponding to the emotions are scored 1 point, and irrelevant answers or incorrect answers are scored 0 points.

#### Appendix A.1.3. E3: Emotional Attribution Interpretation Experiment

This experiment and the emotional viewpoint selection experiment share the same set of situational stories to investigate the children’s understanding of the causes of other people’s emotions. After the researcher conducted an emotional viewpoint selection experiment on the children, they used a calm tone to ask the children the reasons for the emotional state of others in the situation. For example, a child named Coco likes kittens very much. On his birthday, his father brought a kitten back to him. How would Coco feel? The child answered that Coco was very happy. The researcher needs to continue to ask questions, why do you think Coco is happy? The child needs to answer because he received a gift he likes. The scoring standard of the emotional attribution interpretation experiment adopts a two-point scoring method, and the score of the emotional attribution interpretation task is between 0 and 8 points. Participants receive 2 points for correct attributions, 1 point for correct attributions, and 0 points for irrelevant answers or incorrect attributions.

#### Appendix A.1.4. E4: Belief-Based Emotional Understanding Experiment

This experiment adopts the experimental method of Sarti et al. (2019), using the story situation method and the story pictures to tell the situational story for the test children, it investigates the children’s understanding of the characters in the situation based on beliefs and emotions and the reasons for the emotions. In order to ensure the children’s understanding of the situational story, the gender of the characters in the picture is consistent with that of the children, and the expressions of the characters in the picture are blocked. After listening to the situational story, the children need to answer the memory test questions and the fact test questions first. If the answers are correct, ask questions based on beliefs about emotional understanding. If the answer is wrong, the situational story needs to be told to the child again until the child fully understands and answers the memory test and fact test questions correctly. The specific situational story is presented in the appendix. The questions of emotional understanding based on belief are as follows: Coco came back from outside, picked up the cup on the table, and prepared to drink. What was his mood before Coco drank the cup? (Is he happy or unhappy?) Why is there such a mood? The scoring standard of the belief-based emotion understanding experiment uses a 2-point scoring method, which participants who were able to correctly answer that Coco was happy before drinking something in the cup and correctly explained the reason was scored 2 points, only those who were able to answer emotions that could not explain the reason were scored 1 point, and irrelevant answers or incorrect answers were scored 0 points.

#### Appendix A.1.5. E5: Desire-Based Emotional Understanding Experiment

This experiment and the belief-based emotion understanding experiment use the same set of situational stories to investigate the children’s understanding of the situational characters based on desire emotions and the reasons for their emotions. The questions of emotional understanding based on desire are as follows: What is his/her mood after Coco comes back from the outside and drinks something in the cup? (see Appendix A Figure A3) Is he happy or unhappy? Why does he feel this way? The scoring standard for the desire-based emotion understanding experiment uses a two-point scoring method. Participants who were able to correctly answer that they were unhappy after Coco drank something in the cup and correctly explained the reason was scored 2 points, only those who were able to answer emotionally unexplainable reasons were scored 1 point, and irrelevant answers or incorrect answers were scored 0 points.

#### Appendix A.1.6. E6: Conflict Emotional Understanding Experiment

This experiment uses situational stories to create two conflicting emotions in one story and investigates the children’s understanding of the two emotions simultaneously produced in the same event. Researchers tell contextual stories. For example, Coco received a gift from his mother on his birthday, but his mother said that the gift cannot be opened today, and the gift can be opened tomorrow. Coco felt both happy and unhappy. The researcher asked the children the following questions: Why is Coco happy? Why is Coco unhappy? The scoring standard of the conflict emotion understanding experiment uses a 2-point scoring method. Participants who can correctly answer two reasons for emotions are scored 2 points, those who can only answer one reason for emotions are scored 1 point, and irrelevant answers or incorrect answers are scored 0 points.

## Appendix B

**Table A1:** Emotion understanding record sheet.

No.	Sex	Age	Expression Naming				Expression Recognition				Situational Understanding				Situational Attribution				Belief	Desire	Conflict
			1	2	3	4	1	2	3	4	1	2	3	4	1	2	3	4			
**1**																					
**2**																					
**3**																					
**4**																					
**5**																					
**…**																					

**Table A2:** Children’s emotional regulation questionnaire (for parents).

No.	Children’s Specific Performance	Never → Always: 1 → 5				
A. When playing with friends, other children accidentally bump into your child, children will generally:						
1	It is okay. He came across me by accident, not on purpose.	1	2	3	4	5
2	Give him a push at once.	1	2	3	4	5
3	Immediately back away from the child who hit him.	1	2	3	4	5
4	Although some are not happy, but can quickly continue to play their own.	1	2	3	4	5
5	Start crying at once.	1	2	3	4	5
6	Tell the teacher that other children hit people.	1	2	3	4	5
B. When playing with toys, your child’s toys are stolen by other children. It is estimated that he will:						
7	Kindly ask the child for toys or exchange toys with him.	1	2	3	4	5
8	Switch to other toys or other activities.	1	2	3	4	5
9	Push the child with hands or kick with feet, to grab the toys.	1	2	3	4	5
10	Yell or roll on the floor.	1	2	3	4	5
11	Tell the teacher, hope the teacher can help to get back the toys.	1	2	3	4	5
C. Child wants to watch his favorite cartoon, his parents say, “you’ve been watching it for a long time. Don’t watch TV again”. He will:						
12	They turn to other toys or games they like or do other things soon.	1	2	3	4	5
…						

**Table A3:** Children’s resilience assessment scale (for parents).

No.	Items	Never → Always: 1 → 5				
1	Listen to or respect others.	1	2	3	4	5
2	Show confidence in your abilities (e.g., “I can do it”).	1	2	3	4	5
3	Lose temper.	1	2	3	4	5
4	Try again and again when fail.	1	2	3	4	5
…						
